# Centralised or Localised Pathogen Whole Genome Sequencing: Lessons Learnt From Implementation in a Clinical Diagnostic Laboratory

**DOI:** 10.3389/fcimb.2021.636290

**Published:** 2021-05-18

**Authors:** Alicia G. Beukers, Frances Jenkins, Sebastiaan J. van Hal

**Affiliations:** ^1^ Department of Microbiology and Infectious Diseases, Royal Prince Alfred Hospital, Sydney, NSW, Australia; ^2^ Faculty of Medicine, University of Sydney, Sydney, NSW, Australia

**Keywords:** centralised, localised, whole genome sequencing, clinical microbiology, pathogen genomics, public health

## Abstract

Whole genome sequencing (WGS) has had widespread use in the management of microbial outbreaks in a public health setting. Current models encompass sending isolates to a central laboratory for WGS who then produce a report for various levels of government. This model, although beneficial, has multiple shortcomings especially for localised infection control interventions and patient care. One reason for the slow rollout of WGS in clinical diagnostic laboratories has been the requirement for professionally trained personal in both wet lab techniques and in the analysis and interpretation of data, otherwise known as bioinformatics. A further bottleneck has been establishment of regulations in order to certify clinical and technical validity and demonstrate WGS as a verified diagnostic test. Nevertheless, this technology is far superior providing information that would normally require several diagnostic tests to achieve. An obvious barrier to informed outbreak tracking is turnaround time and requires isolates to be sequenced in real-time to rapidly identify chains of transmission. One way this can be achieved is through onsite hospital sequencing with a cumulative analysis approach employed. Onsite, as opposed to centralised sequencing, has added benefits including the increased agility to combine with local infection control staff to iterate through the data, finding links that aide in understanding transmission chains and inform infection control strategies. Our laboratory has recently instituted a pathogen WGS service within a diagnostic laboratory, separate to a public health laboratory. We describe our experience, address the challenges faced and demonstrate the advantages of de-centralised sequencing through real-life scenarios.

## Introduction – Pathogen Whole Genome Sequencing: Current Situation and Future Directions

Outbreaks of multi-drug resistant (MDR) pathogens represent a constant threat to healthcare systems worldwide. Infections with MDR pathogens are associated with higher morbidity and mortality rates than susceptible pathogens due to limited available treatment options (Scott, 2009; Vincent et al., 2009; Gandra et al., 2019). Effective infection control is therefore important for mitigating potential outbreaks.

Pathogen whole genome sequencing (WGS) has been demonstrated as a useful tool for identifying outbreaks and indicating points of intervention (Arnold, 2015; Gilchrist et al., 2015; Brown et al., 2019). WGS has become progressively available due to its increased affordability, which has led to its improved uptake for pathogen WGS in a clinical diagnostic space (Deurenberg et al., 2017; Berry et al., 2020). This technology is constantly evolving with some of the more recent advancements including a more rapid turn-around time of results, especially in relation to technologies such as Oxford Nanopore Technologies (Tyler et al., 2018).

Currently, pathogen WGS follows a centralised sequencing approach modelled off a public health process where isolates are sequenced at a centralised laboratory, analysed and a generic analysis report produced (Grant et al., 2018). Although this model is beneficial, by reducing the costs associated with WGS, the turnaround time can be quite long, effectively reducing the practicality of using WGS data in outbreak investigations. Further, there is disconnect between those requesting the data and those analysing and interpreting it, limiting its useability.

A transition to WGS at a local level would therefore be more suitable for use in a diagnostic laboratory, as data can be generated and analysed in near real-time. Uptake of WGS at a local level, however, has mostly been hindered by the lack of staff with the necessary bioinformatics skills needed for the analysis of data generated. Furthermore, establishment of necessary regulations are still under development to try and standardise this technology across multiple sites.

Here we review our experience with implementing a pathogen WGS service at a localised level in a clinical diagnostic space and demonstrate the benefits of establishing such a service at a local vs a centralised level.

## Centralised vs Localised Sequencing – Shortcomings of a Public Health Model in a Clinical Diagnostic Setting

Improvements in next generation sequencing (NGS) technologies, including development of benchtop sequencing and remote cloud computing and data storage, are helping to support a move to a decentralised model. Our laboratory currently houses an Illumina MiSeq, an Illumina iSeq and more recently an Oxford Nanopore MinION platform. These instruments take up a relatively small benchtop area of between 3.8, 1.0 m^2^ and the size of the computer used to run the MinION platform respectively. Our laboratory started sequencing bacterial isolates in 2017 and since then has undergone a successful accreditation review by the Australian regulatory body; National Association of Testing Authorities, Australia (NATA) in 2019.

**Figure 1:**
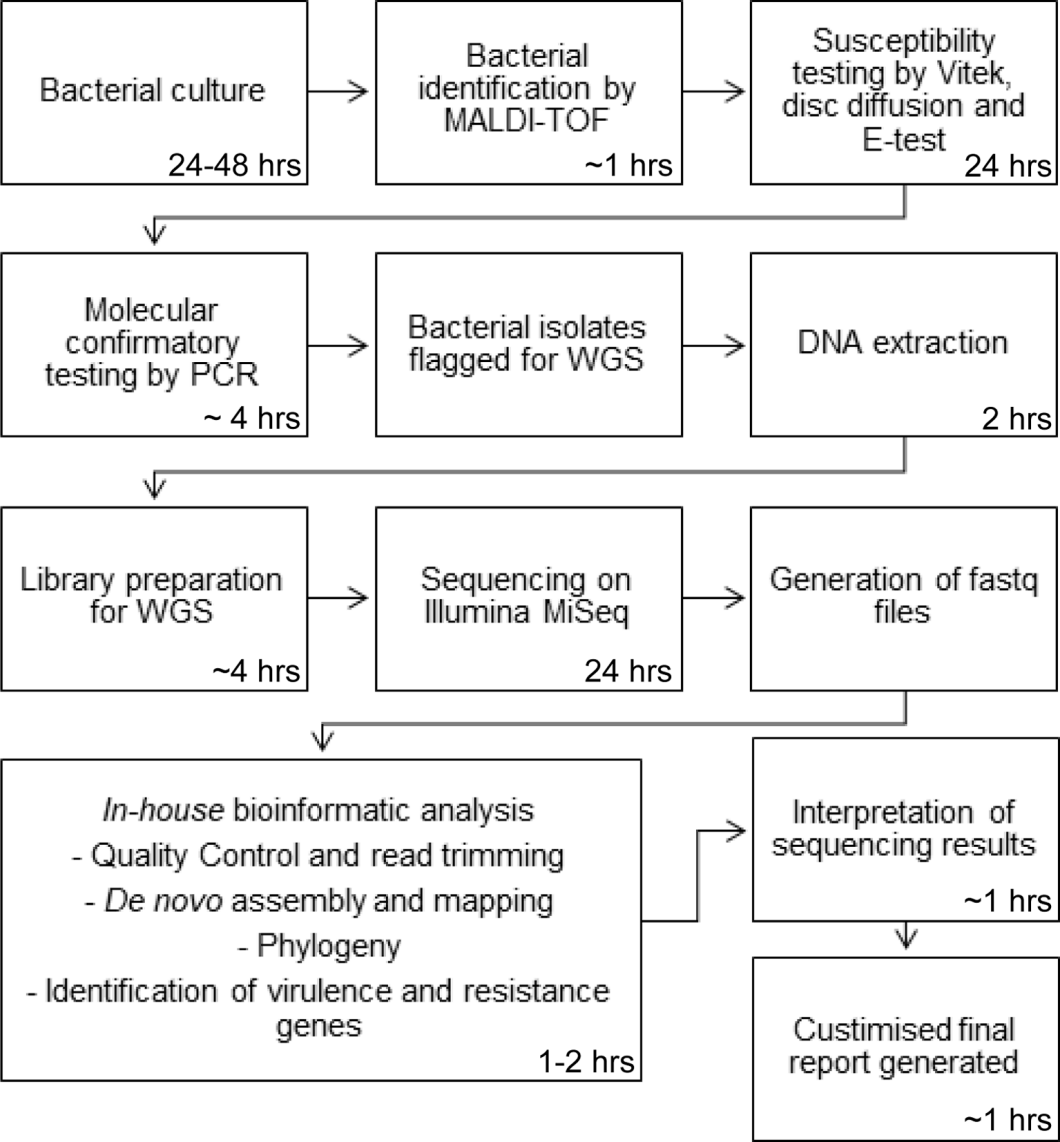
Schematic overview of the laboratory process for WGS.

Our sequencing service currently serves our hospital, a quaternary 900-bed referral hospital that provides several state-wide services, including liver transplantation. Bacterial isolates are sequenced in our laboratory for a number of purposes including outbreak investigations, confirmation of species identification and investigation and confirmation of antibiotic resistance and virulence genes. Sequencing requests are made by our infectious disease specialists with both the wet- and dry-lab procedures undertaken by our microbiologists on site. Our laboratory performs pathogen WGS on approximately 1000 bacterial isolates per year. Pathogen WGS is incorporated into our routine laboratory workflow. In brief, bacterial cultures are first identified by species through matrix-assisted laser desorption/ionization-time of flight (MALDI-TOF) mass spectrometry (MS). Susceptibility testing is then performed using the Vitek and complimented with disc diffusion and E-tests. Further confirmatory testing is performed by our molecular department using PCR for confirmation of vancomycin resistance genes, methicillin-resistant *Staphylococcus aureus* (MRSA), and confirmation of carbapenem resistance genes in Enterobacteriaceae isolates with a raised meropenem MIC. Following WGS, bioinformatic analysis of the samples are performed on site using an *in-house* bioinformatics pipeline by a qualified microbiologist and a customised report generated. A schematic overview of our laboratory process is presented in **Figure 1**. There are several major benefits that are experienced by sequencing at a local level compared to centralisation. First, reduced turnaround time is the most significant improvement as samples do not have to be transported to external sites for sequencing. Moreover, current clinical microbiology practice involves several processes which can take several days for culturing, species identification and susceptibility testing, and weeks for molecular typing. For slow growing bacteria, results can take months to be obtained. WGS can produce all this information in as little as a week (Didelot et al., 2012). Prioritisation of samples at a centralised laboratory is often based on a first-in first-served basis due to the large quantities of samples received which also adds to the turnaround time. At a localised level, samples can be selected based on priority. Fast turnaround times, so the data can be assessed and interpreted in real-time, are especially important in assessing outbreaks, maximising the benefits of utilising WGS technology.

In a centralised laboratory, there is a major disconnect between the samples sequenced and their associated metadata, limiting connections that can be made between isolates. In our laboratory, we undertake a cumulative analysis approach whereby each successive isolate of the same bacterial species is analysed as a group in addition to individual case studies. This allows connections between isolates to be established which may not necessarily be obvious due to large timespans between collection and sequencing. Further, with centralised sequencing and generation of a generic report there is limited ability for interpretation. At a localised level there is a greater degree of communication between clinicians, microbiologist and bioinformaticians allowing shared knowledge and greater understanding of the data generated.

An additional strength of sequencing at a local level is the small number of samples that are considered important. In our setting, a single MRSA from NICU is considered worthy of sequencing with detection of an extended outbreak over time. In a centralised setting, such as in public health, the importance of a single isolate is not recognised and would not warrant sequencing unless a cluster of isolates with suggested links are identified.

The largest bottleneck presented to decentralisation of WGS is the bioinformatics. This is becoming less of an issue as more graphical user-friendly interfaces are being developed (e.g., Galaxy, Illumina’s Basespace). Development of effective semi-automatic pipelines have also allowed those with basic bioinformatics skills to analyse data. However, for a more in-depth or customised analysis, some knowledge of Unix-systems is required in order to utilise the large number of free bioinformatics software packages available. Furthermore, scientific knowledge of the biology of various micro-organisms that are investigated as well as their important genomic features are essential for a comprehensive analysis.

The final obstacle for a decentralised model is development of an accreditation process to standardise testing across multiple laboratory sites. This is still in the development phase, but as more and more laboratories decide to uptake this technology, the accreditation process will become more streamlined and robust. Some of the caveats to accreditation include both wet- and dry-lab parts requiring validation, the methods of WGS are applicable to all microbial species so standardisation needs to be broad and validation of the WGS workflow may have to be performed using a selection of species as opposed to every species. As WGS is far superior to many of the technologies currently used in the laboratory, it can also provide multiple pieces of information such as phylogeny, antibiotic and virulence genes and MLST, with each of these aspects requiring verification.

## Requirements and Regulation of Pathogen Whole Genome Sequencing

NATA is the regulatory body responsible for certifying laboratories across Australia that they comply with regulatory standards and give confidence in the data and reports produced to those seeking their services. A number of criteria are required to be met for a laboratory to receive certification and are based on relevant international standards (e.g., ISO/IEC 17025, ISO 15189, ISO/IEC 17020). The process of accreditation involves a number of steps including first an enquiry followed by an advisory visit, application, document review, assessment and finally accreditation. After the accreditation process is complete, scheduled reassessments and surveillance visits become incorporated into the laboratory process to ensure continued compliance. To help laboratories ensure they comply with NATA, the accreditation process is administered jointly with the RCPA (Royal College of Pathologist Australasia).

WGS has been used routinely in human medical genomics, with standards well established within this field (ISO 15189 Medical Laboratories – Requirements for quality and competence, Requirements for human medical genome testing utilising massively parallel sequencing technologies). Currently, there is no specific guidance regarding quality issues, validation and requirements of supervision using massively parallel sequencing technology in relation to microbial sequencing.

One of the major differences when applying frameworks used in medical genomics is the focus of reporting and sequencing directed at target regions or genes with strict guidelines for the interpretation of mutations. For pathogen WGS, this is less straight forward as the whole genome of micro-organisms are being assessed. The data is somewhat open to interpretation and requires educated judgement for interpreting the results and for making decisions.

As with any other microbiology test demonstrating ongoing quality proficiency is necessary. Although programs exist, these are likewise directed at public health laboratories. A pilot program has been established by RCPA for assessing WGS of infectious agents, with ambitions to continue this program into the future.

Overall, for pathogen WGS to transition to a localised level, it is important that these requirements and regulations become well established. This will assist diagnostic laboratories in the set up and formation of pathogen WGS in their own facilities. It will also ensure the services provided at different locations become standardised, providing more confidence in a decentralised model over a centralised one.

## Benefits of Onsite Sequencing – Case Studies

Pathogen WGS has been routinely performed in our hospital for the last two years. This has been beneficial in a clinical diagnostic space. The reasons for testing can be categorised into three broad categories: patient-centred, hospital-centred and/or investigational.

Patient-centred sequencing directly impacts patient care or outcomes for example, we sequence all *Burkholderia cepacia* complex isolates from respiratory tract samples of cystic fibrosis patients to confirm speciation. These results influence decisions around suitability for lung transplantation.

Similarly, in hotel quarantine, decision about care and release from quarantine are based on SARS-CoV-2 genomics. Our laboratory thus began using WGS sequencing to identify variants of concern (VOC) of SARS-CoV-2 in real-time. To enable a faster throughput and ability to sequence a single isolate, SARS-CoV-2 WGS long-read sequencing is performed using Oxford Nanopore Technologies (ONT) (Bull et al., 2020). One of the major advantages of introducing this technology has been the real time monitoring of genome coverage allowing us to generate a lineage report within 8 hours.

Other patient-centred applications included antimicrobial resistance testing, antiviral resistance testing and metagenomics, although the later remains under investigation. Additional examples of the uses of pathogen WGS are outlined in some of our recent publications listed in **Table 1**.

**Table 1:** Recent publications demonstrating applicability of bacterial WGS in clinical diagnostic microbiology.

Bacteria	Title of study	Investigation type	Methodology	Major findings	Ref
*Staphylococcus aureus*	A multicentre outbreak of ST45 MRSA containing deletions in the spa gene in New South Wales, Australia	Epidemiology, assessment of diagnostic tests	WGS (Illumina MiSeq), read mapping (BWA), SNP calling (freebayes), assembly (SPAdes), phylogeny (treeAnnotator program)	Identified deletion in *spa* gene of *S. aureus* by WGS that inhibited Cepheid Xpert^®^ MRSA/SA BC test therefore informing company of corrective modifications to be made in real time	Beukers et al., 2020
*Escherichia coli, Enterococcus faecalis, Enterococcus faecium, Mycobacterium tuberculosis, S. aureus, Streptococcus pneumoniae, Staphylococcus epidermidis*	Recommendations to address the difficulties encountered when determining linezolid resistance from whole-genome sequencing data	Antibiotic resistance	Bioinformatics, visualisation (CLC genomics Workbench)	Identification of linezolid resistance site (G2576T) in various organisms	Beukers et al., 2018
*Enterococcus faecium*	Relentless spread and adaptation of non-typeable vanA vancomycin-resistant *Enterococcus faecium*: a genome-wide investigation	Epidemiology, outbreak investigation	WGS (NextSeq 500), mapping (BWA; Stampy), SNP calling (freebayes), assembly (SPAdes), MLST, phylogeny (RaxML), mutation rate (BEAST)	Emergence of vanA pstS negative *E. faecium* in NSW with evidence of outbreaks and inter-hospital transmission	van Hal et al., 2018
*Cronobacter sakazakii*	*Cronobacter sakazakii* infection from expressed breast milk, Australia	Epidemiology, outbreak investigation	WGS (Illumina MiSeq), mapping (BWA), SNP calling (freebayes), phylogeny (FastTree)	Epidemiological link established between contaminated expressed breast milk and infant infection with *C. sakazakii*	McMullan et al., 2018
*Enterococcus faecium*	Failure of daptomycin β-lactam combination therapy to prevent resistance emergence in *Enterococcus faecium*	Antibiotic resistance	WGS (Ion Torrent PGM), mapping (CLC Genomics Workbench)	Variable daptomycin MICs associated with the same mutation in *liaF* and *cls* genes	Menon et al., 2018
*Salmonella enterica*, *Klebsiella pneumoniae*	Hospital acquisition of New Delhi Metallo β-Lactamase type-1 (NDM-1) *Salmonella enterica* through inter-species plasmid transmission	Epidemiology, outbreak investigation, horizontal gene transfer	WGS (Illumina MiSeq, Oxford Nanopore Technologies), mapping (BWA), SNP calling (freebayes), assemblies (Skesa, Unicycler), antimicrobial resistance (AMRFinder)	Confirmation of two separate outbreaks; one of *K. pneumoniae* and one of *Salmonella* enterica. Epidemiological links established between two outbreaks with inter-species plasmid transfer of NDM-1 established	Beukers et al.,
*Haemophilus influenza*	Whole genome sequencing identifies opportunistic non-typeable *Haemophilus influenza* rather than a hypervirulent clone	Epidemiology, virulence	WGS (Illumina MiSeq), assembly (SKESA), MLST, mapping (BWA), SNP calling (freebayes), phylogeny (FastTree)	Four cases of invasive non-typeable *H. influenza* infection were demonstrated to be unrelated and with no novel virulence factors	John et al., 2020

**Figure 2:**
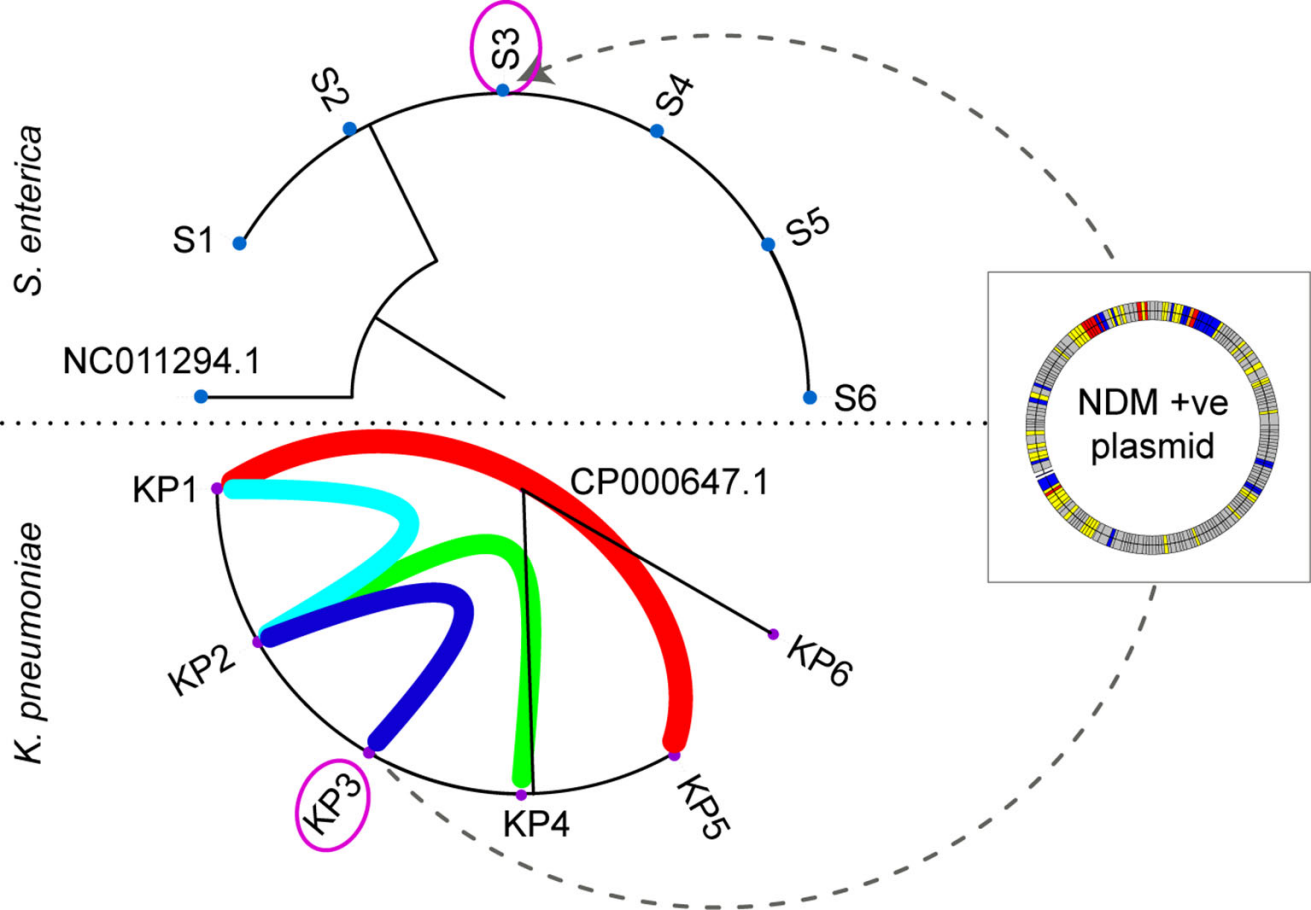
Maximum likelihood phylogeny of the Salmonella enterica (S1-S6) and Klebsiella pneumoniae (KP1-KP6) outbreaks. The purple circled isolate (KP3 and S3) originate from the same patient. The coloured arcs in the K. pneumoniae outbreak demonstrate the epidemiological links between patients, with KP6 confirmed as unrelated to the outbreak. The dotted line between KP3 and S3 indicates the movement of the NDM-1 plasmid from K. pneumoniae and S. enterica within patient 3. The NDM-1 plasmid is depicted in the overlayed box with coloured bands representative of different genes (grey: hypothetical, yellow: mobile genetic elements, red: antibiotic resistance genes, and blue: other).

To highlight how pathogen WGS at a local level has benefited our infection control team (hospital-centred sequencing) we outline an outbreak investigation that spanned over a 17-month period (**Figure 2**). Our laboratory routinely sequences Enterobacteriaceae isolates with a raised meropenem MIC, indicative of carbapenem resistance. From January 2018 until July 2018, our hospital detected several patients colonised with NDM-1 producing *Klebsiella pneumoniae*. Analysis of the sequencing data in conjunction with the epidemiological data provided by our infection control staff we were able to confirm an outbreak. Genomic linkages were also fed back leading to additional reviews of bed movements and exploration of possible “missed” transmission events. With near real-time analysis we were able to direct interventions and identify areas for environmental testing.

Around 7 months after this outbreak was detected, we had increased incidence of *Salmonella enterica* in several patients. WGS combined with epidemiological data, similarly, confirmed *S. enterica* outbreak. Interestingly, one of the patients carried an NDM-1 producing *S. enterica* and had co-located on the same ward as one of the patients involved in the NDM-1 *K. pneumoniae* outbreak. We investigated this further and established inter-species transmission of a plasmid carrying the NDM-1 resistance gene had occurred between *K. pneumoniae* and *S. enterica*.

The connections determined between these two separate outbreaks would unlikely to have been recognised if these isolates were sequenced at a centralised level, considering the timespan that occurred between these two events. As our infectious disease specialists and scientists responsible for performing WGS communicate closely, links between these two outbreaks became more readily apparent and the analysis was able to be modified accordingly.

**Figure 3:**
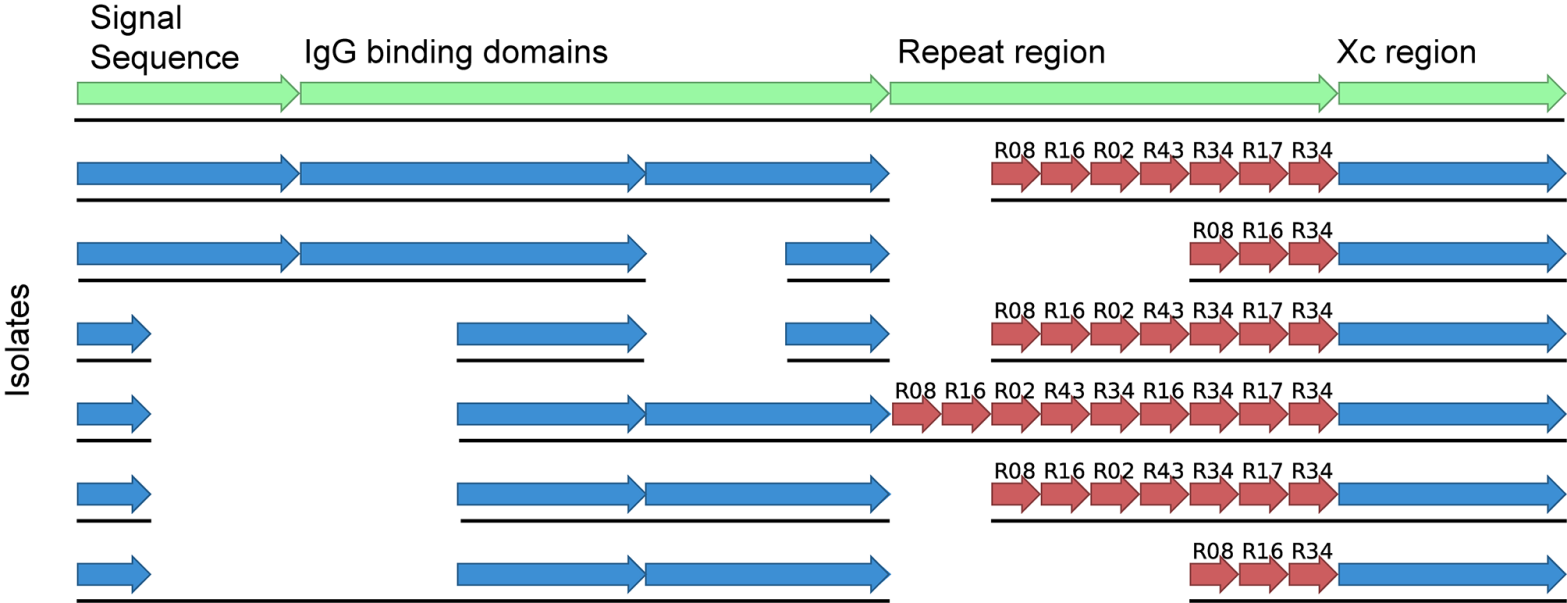
Alignment of the *spa* gene from ST45 MRSA of various *spa* types and indicating the detected deletion event contained within the signal sequence and the IgG binding domains. The first sequence shows the various regions present in the *spa* gene in green whilst the sequences below indicate isolates of different *spa* types with or without the deletion.

Finally, WGS has allowed us to investigate unexpected testing features encountered in the diagnostic laboratory. We noticed an increase in MRSA isolates that were mis-identified as coagulase-negative *S. aureus*. WGS was able to identify a deletion event in the *spa* gene that occurred during the emergence of ST45 MRSA in NSW (**Figure 3**). This deletion made the assay unreliable as a diagnostic test. The company was informed of our results and they were able to modify their diagnostic assay accordingly (Beukers et al., 2020).

## Conclusion

The benefits of onsite pathogen WGS have been clearly demonstrated, with the most advantageous factor of localised compared centralised pathogen WGS an increased turnaround time. As WGS costs continue to decrease and the technology continues to advance, it is becoming more and more feasible for smaller laboratories to implement this technology.

## Author Contributions

AB and SH wrote the initial manuscript. FJ and SH revised the manuscript. All authors contributed to the article and approved the submitted version.

## Conflict of Interest

The authors declare that the research was conducted in the absence of any commercial or financial relationships that could be construed as a potential conflict of interest.
